# Generation of differentially regulated genes in *Mycobacterium tuberculosis* isolates from cerebrospinal fluid and respiratory secretions using suppression subtractive hybridization

**DOI:** 10.1186/1471-2334-14-S3-O16

**Published:** 2014-05-27

**Authors:** SH Saw, VC Yong, YF Ngeow

**Affiliations:** 1 Department of Medical Microbiology, Faculty of Medicine, University of Malaya, Kuala Lumpur-50603, Malaysia; 2 Department of Biomedical Science, Faculty of Science, Universiti Tunku Abdul Rahman, Kampar Pera-31900, Malaysia; 3 School of Biosciences, Taylor’s University Lakeside Campus, Subang Jaya, Selangor Darul Ehsan-47500, Malaysia

## Background

Suppression Subtractive Hybridization (SSH) is an established method for separating DNA molecules that distinguish two closely related DNA samples, which has become a powerful method for generating subtracted cDNA libraries. In this study, we postulated that SSH could be used to identify significant genetic differences between *Mycobacterium tuberculosis* causing meningitis and *M. tuberculosis* causing pulmonary tuberculosis.

## Hypothesis

The unique DNA patterns present only in cerebrospinal fluid (CSF) TB isolates might be markers for neurotropism and neurovirulence in some strains of *M. tuberculosis*.

## Methods

Bacterial DNAs were isolated and SSH was performed using PCR-Select Bacterial Genome Subtraction Kit (Clontech). Twenty subtracted DNAs were generated from 3 CSF isolates and 3 respiratory isolates. Molecular cloning was done on the subtracted DNAs and the positive isolates were extracted, purified and sequenced for analysis.

## Results

So far, 9 of the subtracted DNAs have been successfully cloned into pGEM-T vector, resulting in 21 positive clones. Two preliminary BLASTP results revealed several mycobacterial PE/PPE proteins which play a role in the evasion of host immune responses, possibly via antigenic variation, together with conserved hypothetical proteins with unknown function.

## Conclusion

Further studies are needed to determine the role of these subtracted DNAs.

